# Editorial: Representational states in memory: where do we stand?

**DOI:** 10.3389/fnhum.2015.00453

**Published:** 2015-08-18

**Authors:** Ilke Öztekin, Nelson Cowan

**Affiliations:** ^1^Department of Psychology, Koç UniversityIstanbul, Turkey; ^2^Department of Psychology, University of Missouri at ColumbiaColumbia, MO, USA

**Keywords:** representational states in memory, memory models, working memory, focus of attention, short-term memory, long-term memory

One key debate in memory research centers around the problem of representation. How is information represented in memory and how do the principles that govern memory representations change across the short and long term? One class of memory models (e.g., Baddeley and Hitch, 1974) characterized separate memory stores for information represented over the short-term from those of more permanent and durable long-term representations. More recent approaches (Cowan, 2001; Oberauer, 2002; McElree, 2006) on the other hand, have distinguished memory representations based on distinct activation levels, or states, rather than specialized stores.

For decades researchers have assessed the interactions and dissociations across memory systems and representational states using behavioral investigations, seeking for the key principles that govern them. Recent advances in neuroscience have provided the field with a new set of tools that can be employed to complement and extend previous efforts by means of assessing the corresponding underlying neural mechanisms. In an effort to move toward a more unified perspective, this research topic brought together a collection of empirical, theoretical and review articles that collectively advance our understanding of representational states in memory, as well as bear the potential to reconcile some of the differences across the models.

LaRocque et al. (2014) provide an influential review of the state-based memory models along with an overview of recent efforts to disentangle the hypothesized states utilizing both univariate and multivariate analysis of neural data associated with them. They highlight the inferential advantage of the multivariate approach in providing a more direct test for the critical predictions of the theoretical models due to their sensitivity to the information contained in distributed patterns of neural activity.

Nee and Jonides (2013) propose a descriptive neural account that incorporates the recent neural findings with respect to the neural correlates of distinct representational states. In addition to presumably being a precedent of a computational neural model distinguishing the layers/states of memory, this account provides a reconciliation of some of the discrepancies observed in recent neural findings and those of traditionally held views of short-term memory (STM) and long-term memory (LTM). Most notably, the account attempts to address the involvement of the medial temporal lobes (MTL) for the intermediate representational state (direct access region), between the focus of attention (FoA) and LTM. In this account, MTL is hypothesized to provide the item-context bindings that form the basis of this representational state. They also suggest that MTL-coordinated neural firing during the maintenance of information in STM leads to new LTM.

Based on his theoretical account, Oberauer (2013) has introduced a computational model in an attempt to resolve a key debate in the literature, namely the limit of the current FoA. The computational model is applied to several data sets that have reported inconsistent results. The model shows that some of these discrepancies can be explained by accounting for the specific retrieval operation deployed to access representations from memory, namely whether the memory search is carried out in parallel or serial.

Beaudry et al. (2014) question the necessity of the FoA as a unique representational state in memory assessing a behavioral prediction proposed by some researchers, namely that it should be immune to proactive interference (PI) effects in memory. They review studies in which immunity to PI failed to hold for items residing in the current FoA. They suggest that the relevancy of the contents of focal attention to the task at hand is a key factor for determining whether immunity to PI will be observed. This notable investigation questions the reliance on single measures to understand and distinguish the hypothesized representational states and/or memory systems.

Yee et al. (2014) assess memory performance of amnesic patients on a short-term change detection task that required item-location relationships. This design extends their previous work by ensuring the task demands did not exceed working memory capacity limits. Results from two experiments indicate that amnesic patients were impaired despite a short delay and the absence of intervening items between study and test. A critical question that remains to be tested is whether this impairment is specific to spatial relationships or a more global deficit in relational memory processes.

Morrison et al. (2014) evaluate how memory performance and neural activation indices change across temporal order memory judgments depending on whether the task emphasized earlier or later members of the study list. Primacy and recency effects shifted with the manipulation of the task demands, with larger recency effects observed during blocks of judgments of recency, and larger primacy effects during blocks of judgments of primacy. Their data also complements previous research suggesting that the indices of FoA can change depending on the task demands and the strategy employed. For example, when rehearsal is not possible, FoA usually corresponds to the most recent item on a study list, but might switch across items during rehearsal (McElree, 2006) or serial memory search operations (McElree and Dosher, 1993; Öztekin et al., 2009).

So far, we have focused on findings that relate to accessing episodic (e.g., list membership) information from memory. Elhalal et al. (2014) have introduced a computational account of the role of the prefrontal cortex in semantic relatedness and semantic isolation. Through experiments and model simulations, they show that the magnitude of the Von Restorff effect (better memory for semantic isolates) and semantic clustering were correlated with fluid intelligence, a measure that has been shown to be mediated by the prefrontal cortex. The computational model Categorization-Activation-Novelty (CAN) was able to account for both effects.

We conclude by highlighting several venues for future research. Recent advances in neuroscience now enable powerful approaches that combine behavioral indices along with complementary neuroscience methods that can utilize univariate and multivariate analyses of neuroimaging data on healthy individuals, as well as transcranial magnetic stimulation and lesion studies to test and infer similarities and dissociations across the hypothesized states of memory. The literature would benefit from computational memory models that can account for the recent neural findings (e.g., the contribution of the MTL to both STM and LTM). A more in-depth investigation of the neural correlates of the FoA is also warranted. Recent neuroimaging work has implicated the posterior parietal cortex and the lateral inferior temporal cortex (Öztekin et al., 2010; Nee and Jonides, 2011). Transcranial magnetic stimulation and patient studies could test whether these regions uniquely support successful execution of memory judgments pertaining to the FoA. Future neuroimaging studies could also take advantage of multivariate pattern analysis of distributed neural activity to directly track whether encoded material corresponding to a hypothesized memory state is distinctly represented in the brain. Finally, we should note that verbal and visual working memory studies have yielded inconsistent findings with respect to the capacity limits of FoA (though see Cowan et al., 2014). Future behavioral and neural work investigating the underlying factors for this discrepancy would also be worthwhile.

## Conflict of interest statement

The authors declare that the research was conducted in the absence of any commercial or financial relationships that could be construed as a potential conflict of interest.
